# Generating Flight Illusions Using Galvanic Vestibular Stimulation in Virtual Reality Flight Simulations

**DOI:** 10.3389/fnrgo.2022.883962

**Published:** 2022-04-26

**Authors:** Gaurav N. Pradhan, Raquel Galvan-Garza, Alison M. Perez, Jamie Bogle, Michael J. Cevette

**Affiliations:** ^1^Aerospace Medicine and Vestibular Research Laboratory, Mayo Clinic Arizona, Scottsdale, AZ, United States; ^2^Lockheed Martin Advanced Technology Laboratories, Arlington, VA, United States

**Keywords:** flight illusions, galvanic vestibular stimulation, flight simulation, spatial disorientation, motion perception, virtual reality

## Abstract

**Background:**

Vestibular flight illusions remain a significant source of concern for aviation training. Most fixed-based simulation training environments, including new virtual reality (VR) technology, lack the ability to recreate vestibular flight illusions as vestibular cues cannot be provided without stimulating the vestibular end organs. Galvanic vestibular stimulation (GVS) has long been used to create vestibular perception. The purpose of this study is to evaluate the ability of GVS to simulate common flight illusions by intentionally providing mismatched GVS during flight simulation scenarios in VR.

**Methods:**

Nineteen participants performed two flight simulation tasks—take off and sustained turn—during two separate VR flight simulation sessions, with and without GVS (control). In the GVS session, specific multi-axis GVS stimulation (i.e., electric currents) was provided to induce approximate somatogravic and Coriolis illusions during the take-off and sustained turn tasks, respectively. The participants used the joystick to self-report their subjective motion perception. The angular joystick movement along the roll, yaw, and pitch axes was used to measure cumulative angular distance and peak angular velocity as continuous variables of motion perception across corresponding axes. Presence and Simulator Sickness Questionnaires were administered at the end of each session.

**Results:**

The magnitude and variability of perceived somatogravic illusion during take-off task in the form of cumulative angular distance (*p* < 0.001) and peak velocity (*p* < 0.001) along the pitch-up axis among participants were significantly larger in the GVS session than in the NO GVS session. Similarly, during the sustained turn task, perceived Coriolis illusion in the form of cumulative angular distances (roll: *p* = 0.005, yaw: *p* = 0.015, pitch: *p* = 0.007) and peak velocities (roll: *p* = 0.003, yaw: *p* = 0.01, pitch: *p* = 0.007) across all three axes were significantly larger in the GVS session than in the NO GVS session. Subjective nausea was low overall, but significantly higher in the GVS session than in the NO GVS session (*p* = 0.026).

**Discussion:**

Our findings demonstrated that intentionally mismatched GVS can significantly affect motion perception and create flight illusion perceptions during fixed-based VR flight simulation. This has the potential to enhance future training paradigms, providing pilots the ability to safely experience, identify, and learn to appropriately respond to flight illusions during ground training.

## Introduction

Over the last few decades, technological advancements in the aviation industry have led to enhancements in the maneuverability of aircrafts in flight. Comparatively, over the millions of years of human evolution, our sensory systems developed to maneuver only on the surface of the earth and not in anticipation of flight. As human aviation progressed, this misalliance has manifested in the commonly known problem of spatial disorientation. Physiologically, motion cues are perceived by the vestibular system, which encodes angular and linear acceleration. Our orientation information is processed by the vestibulocerebellar system from the vestibular, visual, and other sensory systems (Davis et al., 2011). Especially with limited ambient visual orientation cues during flight, the inadequacies of our vestibular and other orienting senses in the air can result in orientation illusions. An orientation illusion is an erroneous percept of one's position or motion, either linear or angular, with respect to the Earth's surface. There are many known orientation or flight illusions which can cause spatial disorientation. Specifically, mismatched visual and vestibular information can create flight illusions commonly perceived by pilots, leading to serious safety concerns if a pilot reacts incorrectly while disoriented (Gibb et al., 2011), especially during the absence of adequate ambient visual information. Most aviators report experiencing flight illusions at some point in their careers, with up to a quarter reporting that spatial disorientation has led to a near accident (Pennings et al., 2020). Not only is there increased risk of death or significant injury, but aircrew may also demonstrate increased stress responses (Tornero Aguilera et al., 2020) or reduced performance in completing specific tasks, such as flying an inappropriate flight trajectory during final instrument approach (Boril et al., 2020). Given the lack of physical incidents or repercussions, many of these errors may go underreported.

Aircrew receive significant technical training for flight operations; however, they may be unaware that they have experienced a flight illusion without prior clear identification of illusions in a controlled manner. Effective training on how to counteract illusions and avoid spatial disorientation is key to improving training programs and reducing significant in-flight events (Cheung, 2013). Flight training has been used to prepare pilots for these scenarios, however, in-air training is expensive and not without risk. Various ground-based methods have been used to provide training on flight illusions and spatial disorientation, including didactic lectures, on-ground demonstrations (e.g., using a Bárány chair to demonstrate Coriolis illusion), and advanced, expensive spatial disorientation simulators (Pennings et al., 2020) physically providing rotational and translational motion perceptions. These flight simulation programs have increased in popularity for their effective training methodology and positive transferability to performance in real-world situations (Hays et al., 1992; Blow, 2012). However, the advanced systems are few globally (<1,100) and are mostly used in military and commercial aviation training. The remaining fixed-based simulator systems that also include newer technologies such as VR are less expensive, have smaller footprints and are much more common as they improve the fidelity and immersion of participants in an environment that provides extensive visual information. While these fixed-based training paradigms generally provide high fidelity visual and somatosensory (haptic) inputs, recreating vestibular flight illusions on the ground is challenging for most training programs as vestibular cues cannot be provided without stimulating the vestibular end organs.

In recent years, Galvanic Vestibular Stimulation (GVS), the application of low-level electrical current to the vestibular system to induce the sensation of motion, in synchronization with the visual information in flight simulation, has shown potential to provide users with multimodal sensory perceptions and mitigate simulator-induced motion sickness (Cevette et al., 2012). This work, building on previous uses of GVS applications (Cevette et al., 2012, 2014), evaluated the ability of GVS to simulate common flight illusions by intentionally providing mismatched GVS applications during flight simulation scenarios in VR. Typically, GVS is achieved by passing current in the range of 1–2.5mA, stimulating the vestibular system, which then interprets the GVS-evoked input like an actual head movement (Fitzpatrick and Day, 2004). In one possible GVS setup, yaw perception is achieved by sending current through anodal and cathodal electrodes placed on the mastoid processes behind each ear. Pitch and roll perceptions are created with additional electrodes placed on the forehead and nape of the neck electrodes and sending specific patterns of GVS stimulation (Cevette et al., 2010). Previously, we integrated GVS with a flight simulation program to synchronize visual and vestibular stimulation in near real-time to demonstrate the potential improved simulator immersion with oculo-vestibular recoupling (OVR; Cevette et al., 2012, 2014). This study investigated whether applying GVS mismatched with the visual scene in a flight simulation environment could be a feasible method to help prepare pilots for the vestibular disturbances that often occur in real flight.

We tested two most commonly occurring flight illusions—the somatogravic illusion during take-off and the Coriolis illusions during a sustained turn (Davis et al., 2011). The rapid acceleration during take-off can create a strong backwards tilt perception which can be incorrectly interpreted as a fast pitch up motion known as somatogravic illusion. As the aircraft remains in a steady turn for some time, pitching the head forward causes a vestibular illusion or severe disorientation of a tumbling sensation known as a Coriolis illusion. Both illusions are known to cause spatial disorientation that creates an undue burden on pilots (Klyde et al., 2017). To date, no study has examined whether GVS can recreate these flight illusions with the goal of preparing pilots to appropriately respond to these situations during on the ground training. Our objectives are to examine whether GVS can create these illusions using intentionally mismatching vestibular and visual information, and to assess the impact of mismatched GVS on subjective presence and sickness.

## Materials and Methods

### Subjects

Nineteen participants (Male:Female, 15:4), were enrolled and completed the study protocol approved by Mayo Clinic Institutional Review Board (IRB). Only recruits between 18 and 55 years of age with no history of vestibular disease, migraine, significant balance disorder, or history of severe motion sensitivity were enrolled. A negative urine pregnancy test was required for female participants. Informed consent was obtained from all participants prior to enrollment in accordance with Mayo Clinic's IRB regulations. Participant demographics showed mean values (±std. deviation) of: age (31 ± 9 years), height (1.75 ± 0.1 m), and weight (74.3 ± 19.3 kg).

### Equipment

**Figure 1:**
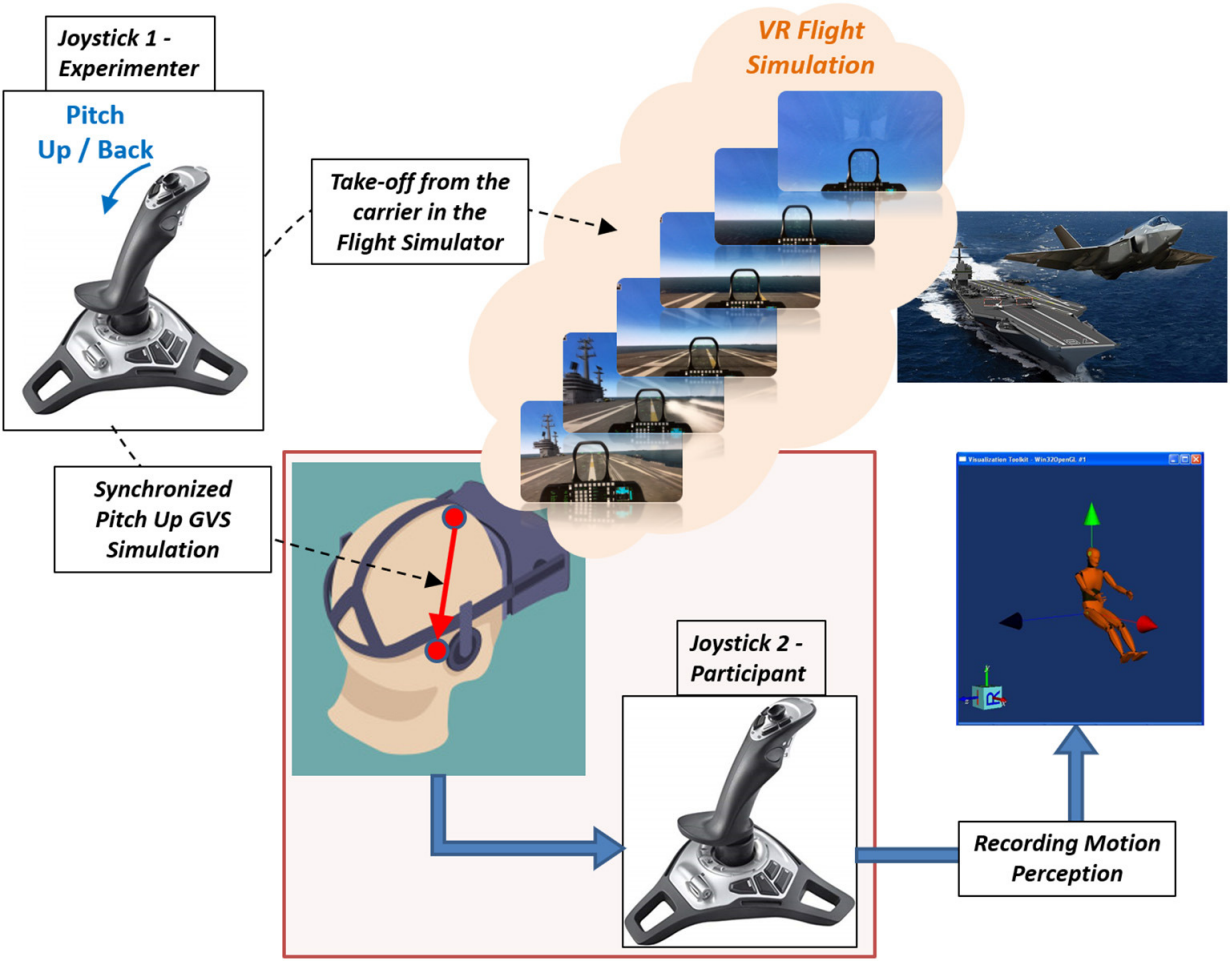
Experimental setup of VR Flight Simulation in the take-off task experienced by the participant. Simultaneously recording of participant's motion perception using Joystick #2 when the experimenter is providing pitch-up GVS commands through Joystick #1 to simulate a somatogravic illusion during a takeoff from an aircraft carrier. Joystick #1's angular movements along the pitch-up axis were transformed into proportional GVS electric currents commands from forehead to mastoid to stimulate pitch-up motion perception.

**Figure 2:**
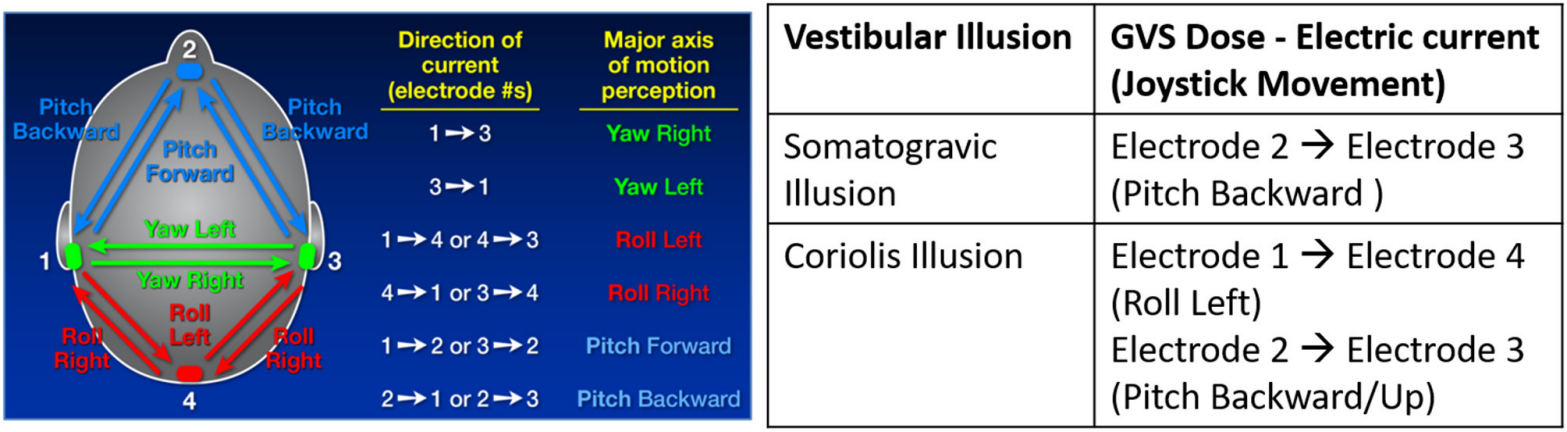
GVS application methodology used to generate motion perception across pitch, yaw and roll axes **(left)** and specific GVS applications to induce somatogravic and Coriolis illusions from joystick movements **(right)**.

Two flight simulation tasks—take off and sustained turn were simulated using Lockheed Martin's Prepare3D® simulation software within the VR environment (Figure 1), using an Oculus Rift CV1 headset. Within the VR environment, the aircraft cockpit was visible to the participants, and simulation tasks were performed during the daytime, in clear sky, over a calm ocean. The participants in a seated position on an arm-rested chair held a three degree of freedom Logitech Freedom 2.4 GHz joystick (Joystick #2) to continuously record their subjective motion perception and flight illusion, if any, during the immersive flight simulation tasks in VR. Joystick #2 was rested on a table in front of the participants. The angular movement of Joystick #2 along all axes (roll, yaw, and pitch) recorded at 5 Hz was converted into corresponding 3D rotational angles to rotate a 3D graphical human figure developed in Visualization Toolkit (VTK; Schroeder et al., 2006) to visualize the participant's perceived motion or illusion. The vestibular stimulation used to create flight illusions was provided by a four-channel galvanic vestibular stimulator (Good Vibrations Engineering, King City, ON, Canada). The visual scene viewed by the participant in VR was duplicated on a desktop computer 2D-screen for the experimenter to see. Another Logitech Freedom joystick was used by the experimenter (Joystick #1). The three-dimensional angular displacement of the experimenter's joystick was inputted to Mayo Clinic's GVS system (Cevette et al., 2010) to generate real-time vestibular stimulation delivered to the participant, creating motion perceptions like those experienced in flight illusions during simulated flight tasks. Figure 2 shows the GVS application methodology (i.e., the direction of current and electrodes involved) to produce motion perception across all axes and the specific GVS application used to induce somatogravic and Coriolis illusions from the joystick inputs (Joystick #1). The GVS system was driven by a proprietary algorithm that transformed the three-dimensional angular displacement of the experimenter's joystick signals into proportional amplitudes and directions of GVS providing expected mismatched motion during flight simulation tasks. The maximum amplitude of GVS was set to 2 mA. The entire range of joystick movement was proportionally matched to the range of electric current from 0 to 2mA for all axes.

### Procedures

#### Take-Off Task (Somatogravic Illusion)

The take-off task in the VR flight simulation program was designed as an automatic catapult take-off from an aircraft carrier ship. It was pre-recorded and shown to the participant in a playback mode within the simulator. The total duration of this task was 40 s. At exactly 10 s into the simulation, the aircraft took off from the carrier and the experimenter simultaneously pulled back the joystick (Joystick #1) to provide a pitch-up GVS signal to the participants with the goal of providing a perception similar to that of the somatogravic illusion. The experimenter was well-trained to provide consistent GVS during flight simulation for all participants. Throughout 40 s of take-off flight simulation, participants were instructed to hold their joystick (Joystick #2) and continuously move it to indicate their perceived motion or illusion if any. The participant's joystick did not control the motion of the aircraft.

#### Sustained Turn Task (Coriolis Illusion)

The sustained turn task in the VR flight simulation program was a long, right banked turn, pre-recorded and shown to the participant in a playback mode within the simulator. The total duration of this task was 60 s. At 30 s into the turn, participants were instructed to pitch their heads forward/down. The experimenter then moved the joystick (Joystick #1) in a simultaneous roll-left and pitch-up motion to generate multi-axis GVS commands in the corresponding directions with the goal of providing a perception similar to that of the Coriolis illusion. At the 45th second, GVS commands were stopped by the experimenter by releasing the joystick to the neutral position. Participants were then asked to move their heads up to the normal upright position. Throughout all 60 s of the sustained turn simulation, participants were instructed to hold their joystick (Joystick #2) and continuously indicate their perceived motion. Again, the participant's joystick did not control the motion of the aircraft.

The experiment occurred in a quiet, climate-controlled room in the Aerospace Medicine and Vestibular Research Laboratory (AMVRL) at Mayo Clinic, Arizona. Participants were asked to consume a light breakfast at least 2 h before the study was started. All participants attended two separate sessions of the experiment on separate days. In one session, participants performed both flight simulation tasks in VR with GVS (“GVS” session) and in the other session, participants performed flight simulation tasks in VR without GVS (“NO GVS”/control session). The order of sessions was counterbalanced across participants to control for training effects such that half of the participants did the GVS session first. Each session was conducted at least 4 days apart to minimize carryover of visual or vestibular effects. The Motion Sickness Susceptibility Questionnaire (MSSQ-Short) was administered to all subjects prior to the first session.

During the “GVS” session, four electrodes were placed on the two mastoids (left and right), forehead, and nape of the neck to deliver the electric currents through the galvanic stimulator. For the “NO GVS” session, four electrodes were placed in the same positions, but the GVS remained off for the duration of the session.

Before putting on the VR headset, participants were trained on how to conduct tasks in the flight simulations on a desktop computer screen (without VR). All participants were given enough time to practice both flight simulation tasks without VR to surpass the learning curve and achieve familiarity with the entire system. They experienced GVS separately along all three axes (roll, yaw, and pitch) to understand how it affects their self-motion perception. They were also trained to indicate the perceived motion along all three axes (roll, yaw, and pitch) with the joystick by showing them the rotating 3D graphical human figure in real-time corresponding to their joystick movement. After training and electrode application, each participant completed flight simulator tasks in VR. During each session, three trials of both take-off and sustained turn tasks were performed. The first two trials of each task were used for experiencing and training within VR purposes, and the last trial was used for further data analysis. The presence questionnaire (PQ) and simulator sickness questionnaire (SSQ) were administered to all subjects immediately after completion of the session.

### Data Analysis

The participants used the 3D angular joystick (Joystick #2) to register motion perception and illusion during the flight simulation experiment. The 3D rotational perception angles along all axes (roll, yaw, and pitch) were proportional to the corresponding angular joystick input. To quantify continuous motion perception, we calculated the following measures from the data streams of the 3D rotational angles along all three axes:

1. *Total Angular Distance:* This variable determines how long and far the participants perceive themselves to be rotated from the initial position.
2. *Maximum Peak Velocity:* This variable determines the intensity of the motion perception in the form of the highest velocity (i.e., rate of change of angular distance) due to angular joystick movement by the participant in perceiving the motion.

An assessment of the normality of these motion perception parameters was performed using the Shapiro-Wilk test. Both parameters violated the assumption of normality (*p* < 0.05) during both flight simulation tasks, and hence, a Wilcoxon signed-rank nonparametric test was used to examine the effect of GVS on inducing flight illusions during flight simulation tasks under two experimental conditions (2 levels—*GVS* and *NO GVS* [control]) in VR. The calculations and acquisition of motion perception parameters from the raw data streams of 3D rotational angles were performed using MATLAB software and the statistical analysis was performed using SPSS software. Subjective simulator sickness and presence during the *GVS* and *NO GVS* sessions were also compared using Wilcoxon signed-rank tests. Spearman's rho correlation was used to examine potential relationships between MSSQ-Short and SSQ scores. All results are expressed as the mean ± standard deviation (SD) unless otherwise specified. Statistical significance was set at *p* < = 0.05.

## Results

### Take-Off (Somatogravic Illusion)

**Figure 3:**
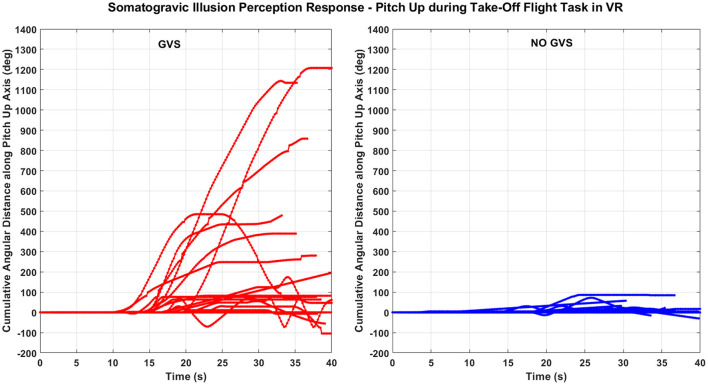
Total angular distance along the pitch axis perceived during the somatogravic illusion while experiencing the take-off task in VR with GVS **(left)** and without GVS **(right)**. Each line indicates an individual participant response. (Few lines short of 40 s indicate participants removing hands from the joystick).

**Figure 4:**
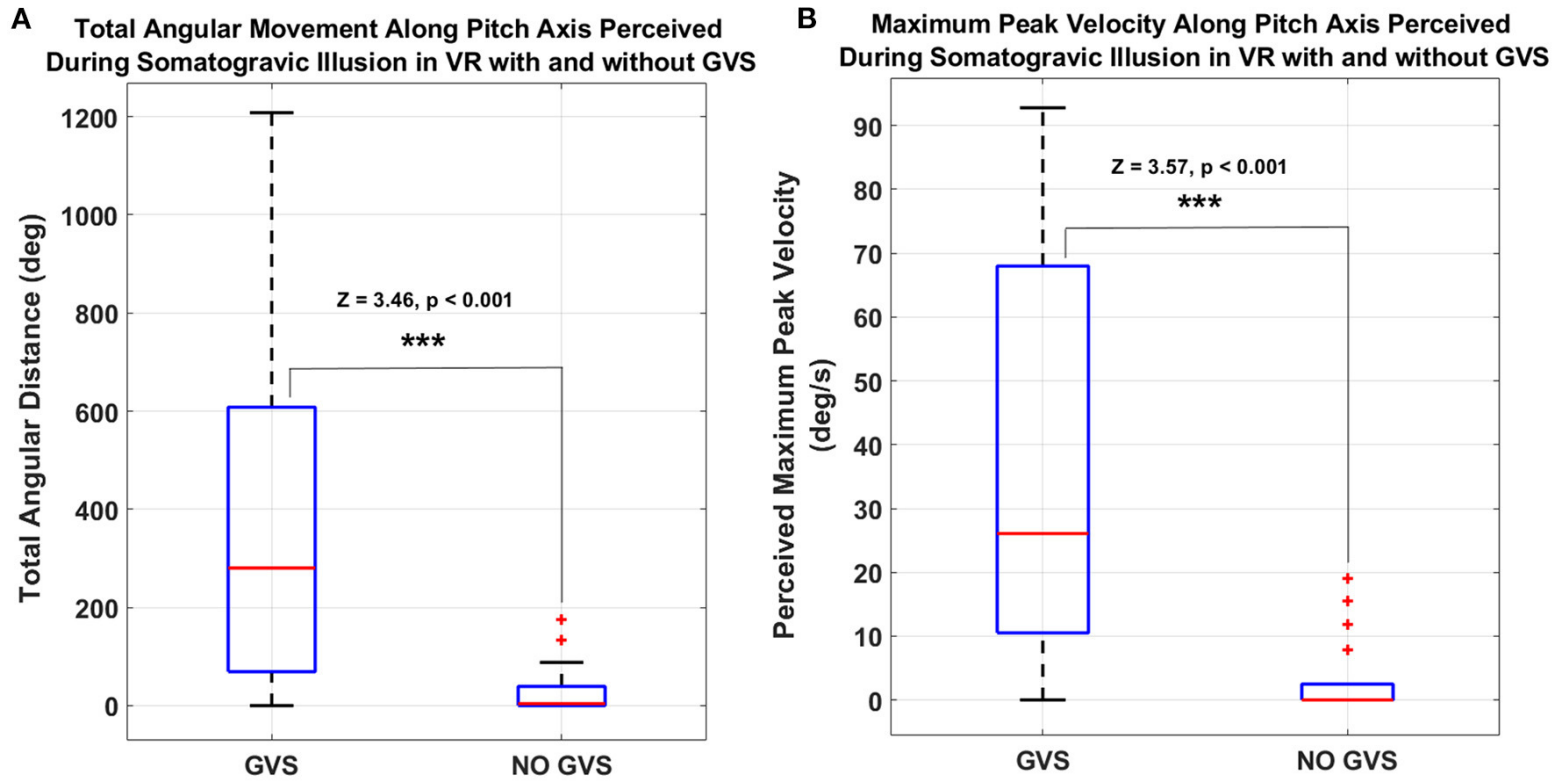
**(A)** Total angular distance and **(B)** peak velocity of participant joystick inputs during the somatogravic illusion take-off task along the pitch axis perceived with GVS and without GVS. (***p < = 0.001. On each box, the central mark indicates the median, and the bottom and top edges of the box indicate the 25th and 75th percentiles, respectively. The whiskers extend to the most extreme data points).

During the take-off flight simulation task in VR, GVS in pitch-up direction was used to induce vestibular disturbance to create the somatogravic illusion (i.e., backwards tilt). Figure 3 shows the comparison of total angular distance along the pitch axis perceived by the same 19 participants during the entire 40 s take-off task with GVS to induce pitch-up motion perception and without GVS. A Wilcoxon signed-rank test showed that for the take-off task, the perceived cumulative angular distance in pitch was significantly larger with *GVS* than *NO GVS* (*Z* = 3.46, *p* < 0.001). The magnitude and variability of the pitch-up motion perception among participants for the *GVS* and *NO GVS* sessions were 390.9 ± 392.3 and 30.1 ± 50.3°, respectively (Figure 4A).

Figure 4B shows the peak velocity of the participants' joystick movements along the pitch axis during the take-off task in the *GVS* and *NO GVS* sessions. The peak velocity of the joystick movements along the pitch axis during the *GVS* session was significantly larger than in the *NO GVS* session (37.1 ± 33.2 vs. 3.2 ± 5.8 deg/s, Z = 3.57, *p* < 0.001, Wilcoxon signed-rank test). The joystick movements in roll and yaw axes during the take-off task were negligible during the *GVS* and *NO GVS* sessions.

### Sustained Turn (Coriolis Illusion)

**Figure 5:**
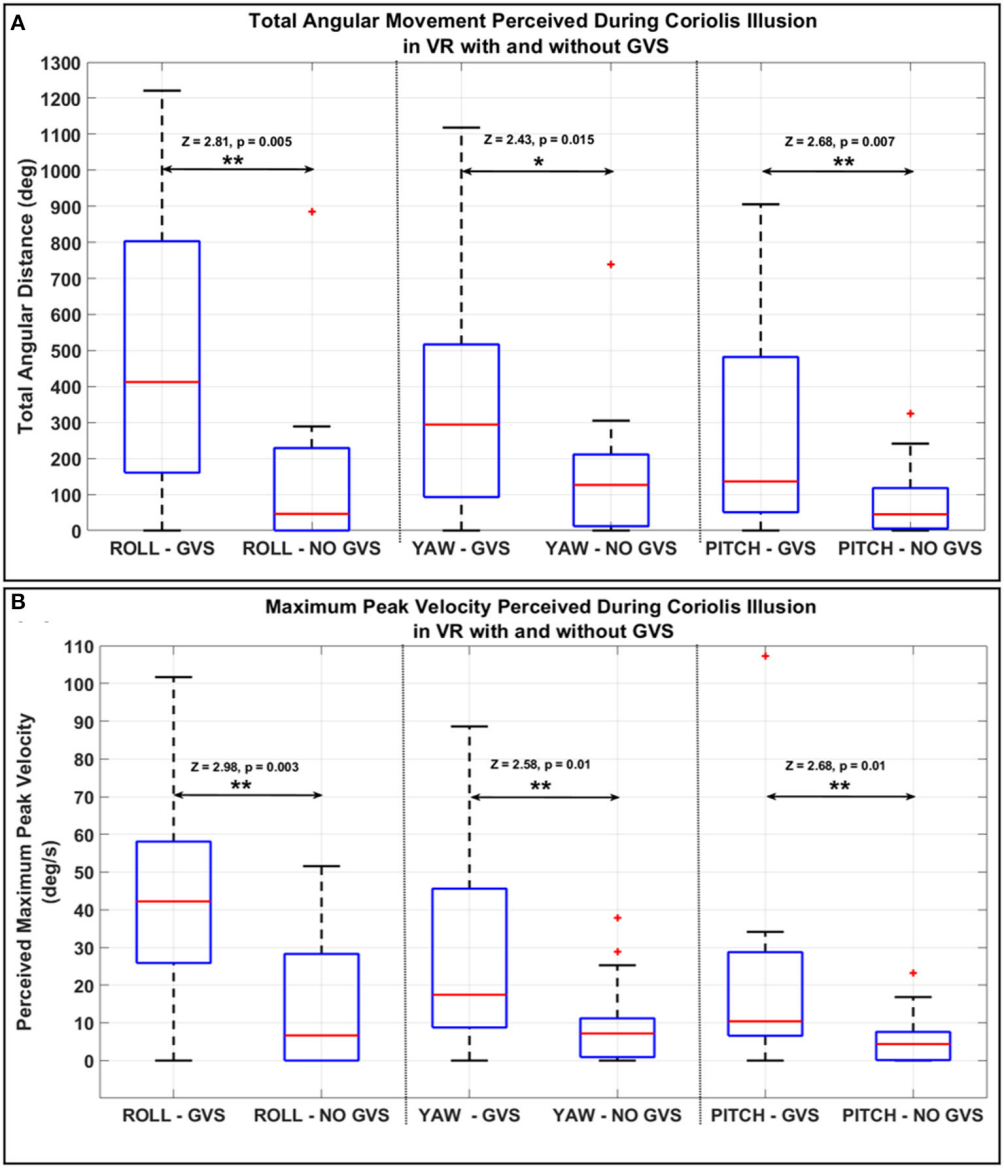
**(A)** Total angular distance and **(B)** peak velocity of the participants joystick along all axes measured during the Coriolis illusion sustained turn task in VR and tilting head forward with GVS and without GVS. (*p < = 0.05; **p < = 0.01. On each box, the central mark indicates the median, and the bottom and top edges of the box indicate the 25th and 75th percentiles, respectively. The whiskers extend to the most extreme data points).

During the sustained turn task (right banked turn) flight simulation task in VR, multi-axis GVS in pitch-up and roll-left direction was applied to induce a vestibular disturbance similar to the tumbling sensation associated with a Coriolis illusion after pitch down head movement. Figure 5A shows the total angular distance along all the three axes, with and without GVS, perceived by the same 19 participants from the time they moved their head forward till the end of the task where they brought their head back to the normal upright position (total 30 s). The total angular distance perceived was significantly larger with *GVS* than *NO GVS* in all axes (roll: median, 412.1 vs. 46.1 deg, *Z* = 2.81, *p* = 0.005); yaw: 293.9 vs. 126.5 deg, *Z* = 2.43, *p* = 0.015; pitch: 136.3 vs. 44.9 deg [median], *Z* = 2.68, *p* = 0.007).

The perceived peak velocities along all axes during the *GVS* session were statistically significantly larger than in the *NO GVS* session (Figure 5B, roll: 43.6 ± 29.5 vs. 13.9 ± 17.6 deg/s, *Z* = 2.98, *p* = 0.003; yaw: 26 ± 24.5 vs. 9.3 ± 10.9 deg/s, *Z* = 2.58, *p* = 0.01; pitch: 19.4 ± 24.1 vs. 5.4 ± 6.5 deg/s, *Z* = 2.68, *p* = 0.007).

### Questionnaire Subjective Ratings

There was no significant difference in PQ scores between the *GVS* (93.8 ± 23.1) and *NO GVS* sessions (88.9 ± 25.8). The total SSQ scores were also not significantly different between the two conditions (*GVS*: 2.9 ± 2.8, *NO GVS*: 2.6 ± 4.3) and were relatively low overall, ranging from 0 to 9 with *GVS*, and 0 to 16 with *NO GVS*, out of a possible 140. However, when split up into Nausea, Oculomotor, and Disorientation categories, the SSQ Nausea score was significantly higher in the *GVS* session (1.9 ± 2.3) than in the *NO GVS* session (1.3 ± 2.5; *Z* = 1.96, *p* = 0.026). While not a primary research question of this study, we were interested in potential correlation between motion sickness susceptibility and presence or simulator sickness reports. There were no significant correlations between the MSSQ-Short (2.3 ± 3.0) and either PQ or SSQ scores for these flight illusion tasks. There were also no significant correlations between PQ and total or category SSQ scores.

## Discussion

Acknowledging the significant operational impact of flight illusions, we investigated whether the application of Galvanic Vestibular Stimulation (GVS) may be a potential method for enhancing training by introducing controlled vestibular cues to recreate vestibular motion perceptions in an accessible way. GVS has long been used to replicate vestibular system perception (Zink et al., 1997; Fitzpatrick et al., 2002). Our previous work found significant motion perception enhancement and reduced simulator sickness with the coupling of visual-vestibular information during flight simulator tasks (Cevette et al., 2012, 2014). In this study, we investigated whether intentionally mismatched GVS could reasonably recreate common somatogravic and Coriolis flight illusions in visual flight simulation scenarios in VR.

Somatogravic illusion can occur in actual flight during a catapult take-off from an aircraft carrier. The fast linear acceleration creates a profound perception of backward/nose-up pitch that could lead to an inappropriate pitch-down response from the pilot. In-flight simulations, visual information regarding this flight scenario can be easily produced with a high level of detail; however, to date there is no vestibular system contribution to this training scenario when using a fixed-based simulator. Figures 3, 4 show minimal pitch-up self-motion perception when the vestibular system was not involved or stimulated, i.e., during the *NO GVS* session. However, the self-reported pitch perception increased 10-fold with the addition of pitch-up GVS (i.e., *GVS* session) during take-off when tested on the same participants (Figures 3, 4). The results from the somatogravic illusion paradigm presented in this study suggest that the application of GVS may be a reasonable method for simulating an approximate somatogravic illusion during ground-based flight training.

The second common flight illusion tested in this study was the Coriolis illusion, a more complex perceptual phenomenon experienced by pilots in conjunction with large head movements under prolonged turns with constant angular velocity (Davis et al., 2011). In this study, during the prolonged right banked turn in VR flight simulation, participants tipped their head down, and the experimenter created a pitch-up, roll-left GVS stimulation to simulate the Coriolis illusion that would be created with this motion in flight. As expected, participants described GVS-induced perceptions in all three planes, with roll as a dominant perception (Figure 5). The significant increase in self-perceived total angular movement and maximum peak angular velocities across all three axes suggest that participants experienced relatively strong and disorientating motion perceptions. As expected with multi-axis stimulation, responses were more variable in terms of degree and direction of perception for the Coriolis illusion task than for the somatogravic illusion task. However, motion perceptions were in line with expectations of overall disorientation and confusion associated with the Coriolis illusion (Holly et al., 2016). Taken together, results suggest that the application of GVS may also be a reasonable ground-based flight training method for simulation of a Coriolis illusion.

Neither subjective presence nor simulator sickness were different with GVS compared to without GVS. When split into categories, the simulator sickness Nausea category was significantly higher with GVS than without, however scores were generally very low, with 42 and 74% of responses being zero (indicating no symptoms of sickness) for the *GVS* and *NO GVS* sessions, respectively. In line with the Nausea results, we expected that these flight illusion scenarios with GVS could cause symptoms of sickness due to causing of disorientation feeling with the application of mismatched GVS. The shortness of the exposures of mismatched GVS likely contributed to the relatively low simulator sickness scores overall. In future studies, questionnaires could be written to ask more specifically about the perception of unexpected or illusory motions. Additionally, participants in this study were not pilots. Subjective presence may differ more if participants had flight experience and could relate their GVS experience to actual flight experience.

The study demonstrated the use of GVS to create motion perceptions like those experienced in two common flight illusions. However, additional illusions (e.g., the leans, graveyard spiral, etc.) should also be considered for future GVS training paradigms. Our current technology limited GVS stimulation to a maximum of 2 mA. As demonstrated by the wide variability in reported perception, it is possible that an expanded stimulation range may be needed to provide more consistent motion cues across trainees. Additionally, we acknowledge the flight illusions often occur in or are made worse in degraded visual environments (DVE) such as clouds or darkness. This study did not include DVE however future studies could examine the impact of DVE on the intensity of GVS-induced illusions.

Most importantly, while we have demonstrated in this and prior studies that we can create vestibular perceptions to enhance motion and/or create illusions, it is unknown if this training paradigm will transfer to actual flight (Baldwin and Ford, 1988). Again, inclusion of experienced aircrew in future work is needed to validate and inform changes to the GVS stimulation so that the resultant motion cues best align with actual in-flight perceptions. It is also possible that even if the GVS induced motion perception is not an exact match to the motion perception experienced in flight, it could still prove valuable in a training paradigm such that pilots can experience a disorientation feeling while doing flight tasks on the ground before going through it in the air. Whether exposure to simulated illusions on the ground would make a pilot more likely to identify and react appropriately to an in-flight illusion remains unknown. There is little data available to demonstrate the effectiveness of simulation training on reducing in-flight errors (Baldwin and Ford, 1988) associated with flight illusions. Pilots, however, do report that these programs are important training components (Pennings et al., 2020), and it stands to reason that providing the pilot with approximate experiences of these illusions on the ground should lead to improved recognition and reaction in the air (Ford and Schmidt, 2000; Landman et al., 2017, 2018).

Humans have not evolved to appropriately interpret motion in the air, and flight illusions continue to be a significant source of risk for aircrew. To our knowledge, this is the first example of using GVS to simulate common flight illusions to enhance future training paradigms. Application of this technique in pilot training could lead to improved reactions to common flight illusions, reducing pilot errors and overall risk.

## Data Availability Statement

The raw data supporting the conclusions of this article will be made available by the authors, without undue reservation.

## Ethics Statement

The studies involving human participants were reviewed and approved by Mayo Clinic Institutional Review Board. The patients/participants provided their written informed consent to participate in this study.

## Author Contributions

GP, RG-G, AP, and MC contributed to the design and conceptualization of the study. GP and JB contributed to the data acquisition. GP, RG-G, and AP contributed to the analysis and interpretation the data. GP, RG-G, and JB contributed to the drafting the article. GP, RG-G, AP, JB, and MC contributed to the revising the manuscript for intellectual content. All authors contributed to the article and approved the submitted version.

## Funding

This study received funding from Lockheed Martin Corporation. The funder had the following involvement in the study: study design, analysis, interpretation of data, the writing of this article, and the decision to submit it for publication.

## Conflict of Interest

RG-G and AP were employed by Lockheed Martin Corporation. The authors declare that the research was conducted in the absence of any commercial or financial relationships that could be construed as a potential conflict of interest.

## Publisher's Note

All claims expressed in this article are solely those of the authors and do not necessarily represent those of their affiliated organizations, or those of the publisher, the editors and the reviewers. Any product that may be evaluated in this article, or claim that may be made by its manufacturer, is not guaranteed or endorsed by the publisher.
